# Association of IFN-*γ* : IL-10 Cytokine Ratio with Nonsegmental Vitiligo Pathogenesis

**DOI:** 10.1155/2015/423490

**Published:** 2015-09-09

**Authors:** Yaswanth Ala, Mohammed Khalid Pasha, Raja Narasimha Rao, Prasanna Latha Komaravalli, Parveen Jahan

**Affiliations:** ^1^Department of Genetics, Osmania University, Hyderabad, Telangana 500007, India; ^2^Central Railway Hospital, Lallaguda, Hyderabad, Telangana 500017, India; ^3^Department of Zoology, School of Sciences, Maulana Azad National Urdu University, Gachibowli, Hyderabad, Telangana 500032, India

## Abstract

*Background and Objectives*. Cytokines regulate immune response and inflammation and play a crucial role in depigmentation process of vitiligo. The present study aimed to estimate the serum levels of pro- and anti-inflammatory cytokines, IFN-*γ* and IL-10, and their ratios in nonsegmental vitiligo patients and healthy individuals from India.* Methods*. Blood samples were collected from 280 subjects and serum IFN-*γ* and IL-10 levels were measured using standard ELISA.* Results*. Nonsegmental vitiligo patients showed increased levels of IFN-*γ* (12.4 ± 3.2 versus 9.9 ± 4.4 pg/mL) and decreased levels of IL-10 (9.3 ± 1.7 versus 11.5 ± 5 pg/mL) compared to controls. Ratio of IFN-*γ* : IL-10 differed significantly from patients to controls (*p* < 0.05). IFN-*γ* concentrations and IFN-*γ* : IL-10 ratio varied significantly with respect to clinical variants, disease stability, and social habits (smoking and alcohol consumption) and showed a positive correlation with disease duration. Family history of vitiligo was significantly associated with IFN-*γ* : IL-10 ratio but not with their individual levels.* Conclusion*. The ratio of IFN-*γ* : IL-10 serum levels may be considered as one of the promising immunological markers in nonsegmental vitiligo. This is the first study considering multiple aspects in relation to ratio of cytokine levels. Similar studies with large samples are warranted to confirm our observations.

## 1. Introduction

Vitiligo is defined as an acquired cutaneous complex disorder resulting from functional loss of melanocytes in epidermis, characterized by milky white macules of various sizes and shapes that tend to enlarge peripherally in the course of time [1]. It is an inflammatory disorder associated with increased expression of inflammatory cytokines in the skin and blood [2]. The melanocytes are targeted by multiple aggressions leading to marked reduction and destruction of pigment cells in vitiligo patients [3]. The prevalence of the disease is estimated to be <0.1% to >8% of the world population and is found to be 0.5–2.5% in India with a high prevalence of 8.8% in Gujarat and Rajasthan states [4, 5]. The pathogenesis of vitiligo includes both intrinsic defects within melanocytes that activate cellular stress response and the autoimmune mechanisms targeting the melanocytes involving both humoral and cell mediated immunity [5–7]. Infiltration of cytotoxic T cells in perilesional lesions is the characteristic hallmark of vitiligo [8]. There is growing evidence that cytokines are important in the depigmentation process and show a cytokine imbalance in the skin of vitiligo patients suggesting their prominent role in autoimmune pathogenesis [9, 10]. Systemic biological therapies used for treating psoriasis and other autoimmune diseases by targeting cytokines indicate that a similar approach might be effective for vitiligo [6].

Cytokines are protein molecules that include Interferons (IFNs), Interleukins (ILs), various Colony Stimulating Factors (CSFs), and Tumor Necrosis Factors (TNFs) which are key molecules in mediating inflammatory and cytokine reactions. Their response due to imbalance or deficiency in the cytokine network may largely determine autoimmune disease susceptibility and severity. Alteration in the concentration of various proinflammatory and anti-inflammatory cytokines such as IL-6, IL-8, IL-10, IL-2, TNF-*α*, and IFN-*γ* has been associated with various autoimmune disorders [11–13]. IL-10 is a potent regulator of anti-inflammatory immune responses and is hence considered to be a downregulator of cytokine production by Th1 cells and macrophages [14, 15]. On the other hand, elevated concentrations of IFN-*γ* initiate apoptosis and it has been suggested that melanocyte death is mediated by apoptosis in the context of autoimmunity [16]. In view of the above literature, we aimed to assess the balance between the proinflammatory IFN-*γ* and anti-inflammatory IL-10 cytokine serum levels in NSV patients and healthy controls from India.

## 2. Materials and Methodology

The present study enrolled a total of 280 subjects that included 130 patients (mean age 27.6 ± 6.9 years) and 150 healthy controls (mean age 26.9 ± 5.6 years) from Central Railway Hospital, Hyderabad, India, in the period of April 2012 to March 2014. Approval for the study was obtained from the Institutional Ethical Committee, Osmania University, Hyderabad, India. The subjects were enrolled after providing informed consent and detailed information was procured from all the subjects regarding demographic, clinical, and family history of vitiligo. The patients enrolled were either on no drug treatment or in the washout period of three months.

Information was recorded in a pro forma with respect to clinical presentation of the disease, age, gender, age at onset, family history, dietary habits, occupation, socioeconomic background, associated diseases, and clinical parameters. Five millilitres (mL) of blood sample was collected from each of the enrolled patients and controls. An inclusion criterion of patients was presence of nonsegmental vitiligo without any other associated disorder as well as not being under any treatment. Patients with segmental vitiligo, known allergies, other skin diseases, or other autoimmune disorders such as Hashimoto's thyroiditis, Graves' disease, type 1 insulin-dependent diabetes mellitus, Addison's disease, psoriasis, rheumatoid arthritis, and thyroid dysfunction which are known to have altered levels of cytokines were excluded from the study [11, 17].

Based upon the spreading of the lesions or white macules at the time of sample collection, the patients were categorized into active vitiligo (existing lesions are spreading and/or new lesions had appeared within the previous six months) and stable vitiligo (no increase in the size or number of lesions within 6 months or more) [18]. The duration of the disease was considered as the time between initial appearance of the clinical symptoms of the disease and sample collection.

### 2.1. Determination of Serum IL-10 and IFN-*γ* Levels

Analysis was performed on blood serum using sandwich ELISA method (eBioscience Inc., San Diego, CA, USA). In brief, the antibody was coated onto a 96-well plate by adding 100 *μ*L of capture antibody (1X) to each well and incubated overnight at 4°C. The wells were blocked with 200 *μ*L/well of 1X assay diluent and incubated for 1 hour at room temperature (RT). After that, 100 *μ*L/well of serum samples and standards was added to the wells and incubated for 2 hours at RT. After incubation, detection antibody (100 *μ*L/well) was added and incubated for 1 hour. Then 100 *μ*L/well of avidin-HRP (Horseradish Peroxidase) was added and incubated at RT for another 30 minutes. The plate was incubated with substrate (100 *μ*L/well) for 15 minutes at RT followed by (50 *μ*L/well) stop solution. Each of the above steps was interspersed by 5–7 washes to ensure no carryover and absorbance was measured at 450 nm and 540 nm. The concentrations were calculated using MPM software version 6.1.

### 2.2. Statistical Analysis

Data was analysed using descriptive statistics to calculate the percentages, mean values, and standard deviation. Student's “*t*-” test, one-way ANOVA, and Pearson correlation were carried out to analyse the variation between patients and controls, clinical variants of vitiligo, and association among the variables, respectively. A *p* value less than 0.05 was considered as statistically significant.

## 3. Results

A total of 130 vitiligo patients with a mean age of 27.6 ± 6.9 years and 150 healthy controls with a mean age of 26.9 ± 5.6 years were enrolled in this study. Approximately, equal numbers of males and females were observed in both of the study groups. The mean age at onset of the disease was 23.3 ± 7.0 years (23.2 ± 7.8 years in females and 23.4 ± 6.5 years in males) and the duration of the disease ranged from 1 to 14 years with a mean of 4.2 ± 3.1 years (4.6 ± 3.5 years in females and 3.8 ± 2.8 years in males). In the present study 63.8% of the patients showed active vitiligo and 36.2% stable vitiligo; family history of NSV was seen in 40% of patients and 32% of male patients had the social habits of smoking and alcohol consumption (Table 1).

### 3.1. IFN-*γ* Serum Concentrations

The proinflammatory cytokine IFN-*γ* levels were noted to be significantly elevated in vitiligo patients compared to healthy controls (12.4 ± 3.2 pg/mL versus 9.9 ± 4.4 pg/mL; *p* < 0.05). Analysis of variance (ANOVA) showed a significant difference with respect to serum IFN-*γ* levels among the clinical variants of vitiligo (nondermatomal, acrofacial, mucosal, and focal) (*p* < 0.05). Increased amounts of IFN-*γ* were observed in active vitiligo patients compared to patients with the stable condition of the disease (*p* < 0.05). With respect to social habits (smoking and alcohol consumption), the cytokine levels were analysed for only male patients and controls as none of our female subjects were with these social habits.

**Figure 1:**
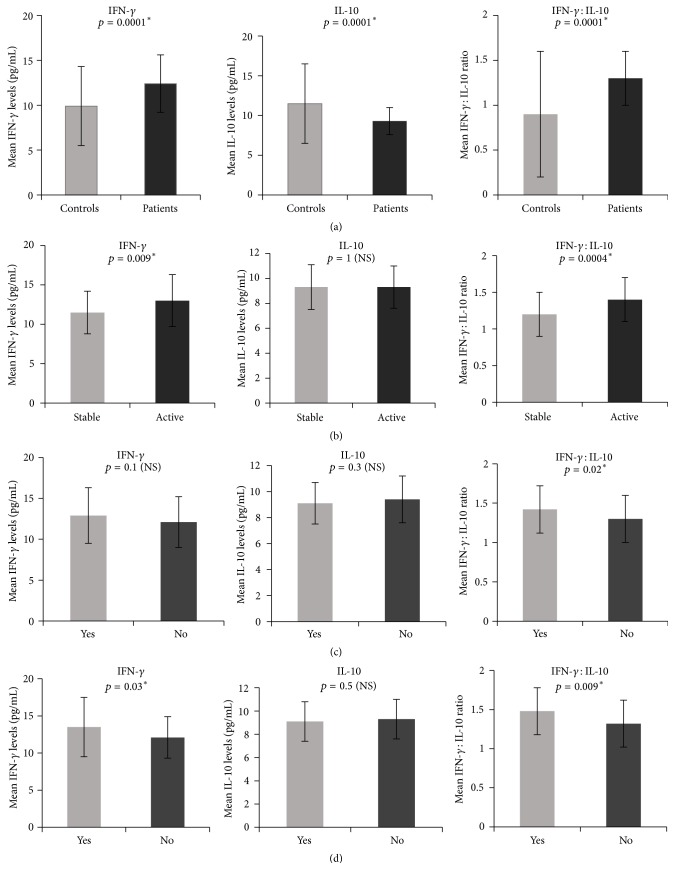
Representing mean IFN-*γ*, IL-10, and IFN-*γ* : IL-10 ratio in (a) patients and controls, (b) patients with active and stable vitiligo, (c) patients with family history of vitiligo, and (d) patients with a habit of smoking and alcohol consumption.

**Figure 2:**
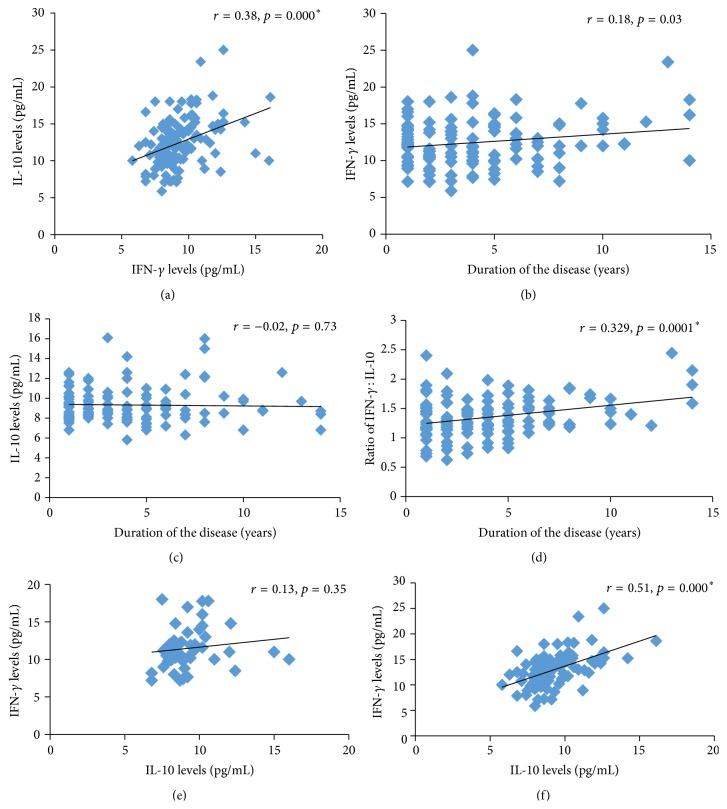
Representing correlation of (a) IL-10 and IFN-*γ* levels in patients, (b) duration of the disease with IFN-*γ* levels, (c) duration of the disease with IL-10 levels, (d) duration of the disease with IFN-*γ* : IL-10 ratio, (e) IL-10 with IFN-*γ* levels in stable vitiligo patients, and (f) IL-10 with IFN-*γ* levels in active vitiligo patients.

Significantly elevated concentrations of IFN-*γ* were noticed in patients compared to controls (*p* = 0.001) and in patients with social habits compared to patients without social habits (*p* = 0.04). However, the levels were not significant between controls with and without social habits (*p* = 0.156). IFN-*γ* levels were nearly similar in patients with and without family history of vitiligo (*p* > 0.05) (Table 2) (Figure 1). A positive correlation was observed between the concentration of this proinflammatory cytokine and the duration of the disease (Figure 2).

### 3.2. IL-10 Serum Concentrations

A significant difference was found in the mean serum concentrations of the anti-inflammatory cytokine IL-10 between patients and controls (9.3 ± 1.7 pg/mL versus 11.5 ± 5 pg/mL; *p* < 0.05). However, no difference in the levels of IL-10 with respect to clinical variants, disease stability, social habits, and family history within the patient group was seen (*p* > 0.05) (Table 2, Figure 1). Furthermore, we did not find correlation between the duration of disease and serum levels of IL-10 (*p* > 0.05) (Figure 2).

A positive correlation between the concentrations of the two cytokines IL-10 and IFN-*γ* was seen in patients but not in controls. Similarly, active vitiligo patients exhibited a positive correlation between IL-10 and IFN-*γ* but the stable vitiligo patients did not (Figure 2).

### 3.3. Ratio of IFN-*γ* : IL-10 in Serum

The ratio of IFN-*γ* to IL-10 was detected to be significantly higher in NSV patients compared to controls (1.3 ± 0.3 versus 0.9 ± 0.7; *p* < 0.05). Patients with clinical variants, with active and stable vitiligo, with and without social habits of smoking and alcohol consumption, and with and without family history of nonsegmental vitiligo also exhibited a significantly increased ratio of IFN-*γ* : IL-10 (*p* < 0.05) (Table 2, Figure 1). Further, there was a positive correlation between this ratio and the duration of the disease (*p* < 0.05) (Figure 2).

## 4. Discussion

In the present study, the serum concentration of the proinflammatory cytokine IFN-*γ* was significantly elevated in NSV patients compared to controls. Supporting our observation, Th1 predominance has been reported to be associated with vitiligo from previous studies. Shi and Erf in Smyth line (SL) chicken model for autoimmune vitiligo have shown that IFN-*γ*, a proinflammatory cytokine which acts as a signature cytokine of Th1 mediated immunity, has remarkably increased in the Smyth line vitiligo samples proving the central role of this cytokine in the SLV pathomechanism [19]. Rashigi et al. reported that both vitiligo patients and mouse model of vitiligo reflect a uniquely IFN-*γ* specific Th1 cytokine signature in the skin that includes IFN-*γ* dependent chemokines such as CXCL9, CXCL10, and CXCL11 which induces T cell homing into peripheral tissues [6]. Mechanistic studies in mouse model revealed that depigmentation requires IFN-*γ*, which induces the local accumulation of melanocyte specific CD8^+^ T cells within the skin supporting the critical role of IFN-*γ* in vitiligo [20]. Further, Nigam et al. also reported an increased number of CD8^+^ T cells in patients with vitiligo compared to controls [21]. Gregg et al. suggested that vitiligo is entirely dependent on CD8^+^ T cells, while CD4^+^ T cells exert a negative regulatory effect and the genetic ablation of IFN-*γ* resulted in scarce CD8^+^ T cell infiltration into the skin [22]. Skin explant model studies reported that the stronger the CD8^+^ T cell response the more elaborate the vitiligo [8]. IFN-*γ* indirectly increases the expression of intercellular adhesion molecule-1 (ICAM-1) on melanocytes and enhances T cell-melanocyte attachment in the skin and thus establishes a link between cytokine and T cell mediated destruction of melanocytes in vitiligo [23, 24].

Decreased serum concentrations of the anti-inflammatory cytokine IL-10 were observed in patients compared to controls suggesting low Th2 cytokine profile in the pathogenesis of vitiligo. Shi and Erf suggested that the physiological inducer of T regulatory cells (Tregs) function and proliferation, IL-10 cytokine, was found to be decreased in active vitiligo lesions [19]. Taher et al. and Tembhre et al. had reported increased levels of this immunosuppressive cytokine in vitiligo patients who showed the repigmentation process upon treatment with tacrolimus and narrow band ultraviolet B (NB-UVB), respectively [17, 25]. This indicates that upregulation of IL-10 may be responsible for the drug response which indirectly supports the decreased levels of IL-10 in vitiligo pathogenesis observed in our study.

Contrary to the serum levels observed at systemic level in the present study, Grimes et al. reported increased IL-10 mRNA levels in involved and uninvolved tissue of vitiligo [26]. Estimation of the cytokines in the involved tissue and circulating levels together in the same patients and comparison with normal healthy controls may help in understanding the discrepancy in the results.

The higher IFN-*γ* : IL-10 ratio noted in our patients compared to controls corroborates the cell based studies of Dwivedi et al. and Nigam et al. from India who observed a decrease in the ratio of CD4^+^/CD8^+^ T cells in vitiligo patients compared to controls signifying the role of T cell mediated pathogenesis in vitiligo [18, 21]. The impaired cytokine network might contribute to the reduction and loss of functional Tregs which are involved in immune tolerance [27]. This implies that the balance between pro- and anti-inflammatory cytokines may play an important role in the pathogenesis of NSV.

Another significant observation of the present study was increase in the anti-inflammatory cytokine with an increase in the proinflammatory cytokine exhibiting a positive correlation between IL-10 concentrations and the IFN-*γ* levels in NSV patients indicating that there might be a concomitant increase in IL-10 and IFN-*γ* levels at the individual patient level. Shi and Erf showed a strong association of IFN-*γ* with a parallel increase in IL-10 suggesting the Th1 polarization in Smyth line chickens. The amount of the anti-inflammatory cytokine IL-10 might have increased to be a counterpart of the proinflammatory effect which could be insufficient to control the proinflammatory cascade of events that are responsible for melanocyte destruction [19].

The basis behind various clinical presentations of NSV such as nondermatomal, acrofacial, mucosal, and focal variants at the time of diagnosis is not well understood. In the present study, evaluation of serum IFN-*γ* levels and the ratio of IFN-*γ* : IL-10 among clinical variants of NSV exhibited a significant difference. However, no difference was observed in relation to serum concentrations of IL-10 alone. Acrofacial vitiligo showed the highest IFN-*γ* and the least IL-10 concentrations among the variants. These observations may gain support by the findings of Gupta et al. and Shah et al. who stated that acrofacial and nondermatomal vitiligo conditions are very difficult to treat [28, 29], which indirectly indicates the relation between higher proinflammatory environment and clinical presentation. The ratio of IFN-*γ* : IL-10 may reflect the state of inflammation in NSV pathogenesis. However, in order to support our observation, studies with a large sample size in relation to clinical variants should be taken up.

In addition, the data was evaluated by taking the duration of the disease into consideration, which showed a positive correlation with IFN-*γ* and IFN-*γ* : IL-10 ratio but not with IL-10 levels. It gives a hint that systemic proinflammatory environment may increase with increase in the duration of the disease. Due to lack of literature in this aspect, we claim indirect support from another study that showed higher grades of response to UVB treatment in patients with recent vitiligo compared to those with long-standing disease [30].

Appraisal of proinflammatory and anti-inflammatory cytokines in relation to active and stable vitiligo revealed significantly elevated amounts of IFN-*γ* and IFN-*γ* : IL-10 ratio and no variation in the serum concentrations of IL-10. Similar to our observation, another Indian study has also reported higher IFN-*γ* serum concentrations in active vitiligo compared to the stable one [23]. Further, our results are supported by the cell based studies which showed an increased number of CD8^+^ T cells compared to CD4^+^ T cells in the blood samples of active vitiligo compared to the stable one. Our observation of elevated IFN-*γ* : IL-10 ratio in the former group compared to the latter also goes hand in hand with the report of Dwivedi et al. showing decreased ratio of CD4^+^/CD8^+^ count [18]. Further,* in vitro* direct analysis of margins of vitiliginous skin showed polarized type 1 T cells (CD4^+^ and particularly CD8^+^) that predominantly secrete IFN-*γ* and TNF-*α* cytokines that are associated with the destruction of melanocytes during active vitiligo [31].

Significantly elevated concentrations of IFN-*γ* and the ratio of IFN-*γ* : IL-10 in male patients with social habits suggest smoking and alcohol may contribute to increased inflammatory response. Smoking and alcohol consumption are known to affect the balance between oxidants and antioxidants [32, 33].

Six to thirty-eight percent of patients with this complex disorder were reported to be associated with family background of vitiligo [34]. The present study was comprised of 40% of the patients with the familial history of NSV. Hence, it was felt that the analysis should be carried out taking this aspect into consideration in relation to cytokines. Interestingly, we found that the patients with familial history of NSV exhibited significantly elevated concentrations of IFN-*γ* : IL-10 ratio compared to those without. Further, there was a lack of variation with respect to individual levels of IFN-*γ* and IL-10 between the two subgroups of patients. There are no reports available in the literature in alliance with cytokine levels in vitiligo patients with familial background of vitiligo. We took an initiation to correlate the link between familial history and cytokine levels and their ratio. Based on our present observations, it can be explored towards the potentiality of the ratio as an immunological marker for identifying the high risk individuals from unaffected sibs of NSV patients. In addition, tracing the cosegregation of genetic variants of IFN-*γ* and IL-10 through family studies may provide the role of these cytokines in NSV pathogenesis.

In conclusion, further studies assessing other pro- and anti-inflammatory cytokines and their ratios at systemic as well as epidermal cytokines (lesional and perilesional) before and after the treatment with a large sample size in various ethnic groups are warranted to confirm our results and to open up the new therapeutic options.

## Figures and Tables

**Table 1:** Demographic and clinical characteristics of subjects included in the study.

	Patients (*n* = 130) Mean ± SD	Controls (*n* = 150) Mean ± SD	*p* value
Age in years	27.6 ± 6.9 years	26.9 ± 5.6	**0.34 (NS)**
Males	27.3 ± 6.7 (76)	27.2 ± 6.0 (85)	**0.92 (NS)**
Females	27.9 ± 7.3 (54)	26.3 ± 4.7 (65)	**0.15 (NS)**
Duration of the disease	4.2 ± 3.1 years	NA	
Males	3.8 ± 2.8 years		
Females	4.6 ± 3.5 years		
Age at onset	23.3 ± 7.0 years	NA	
Males	23.4 ± 6.5 years		
Females	23.2 ± 7.8 years		

	*n* (%)	*n* (%)	

Males with social habits			
Yes	29 (22.3)	20 (23.5)	
No	47 (77.7)	65 (76.5)	
Clinical variants of vitiligo			
Nondermatomal	58 (44.6)	NA	
Acrofacial	33 (25.4)	—	
Mucosal	22 (17)	—	
Focal	17 (13)	—	
Disease status			
Stable	47 (36.2)	NA	
Active	83 (63.8)	—	
Family history			
Yes	52 (40)		
No	78 (60)	—	

Note: NS: not significant; *n* = number of subjects, and SD = standard deviation.

**Table 2:** Serum concentrations of IFN-*γ*, IL-10, and IFN-*γ* : IL-10 ratio in controls and vitiligo patients.

	IFN-*γ* in pg/mL	IL-10 in pg/mL	IFN-*γ* : IL-10 in pg/mL
	Mean ± SD	Mean ± SD	Mean ± SD
Vitiligo patients (130)	12.4 ± 3.2	9.3 ± 1.7	1.3 ± 0.3
Controls (150)	9.9 ± 4.4	11.5 ± 5.0	0.9 ± 0.7
“*t*-” test *p* value	0.0001^**∗**^	0.0001^**∗**^	0.0001^**∗**^
Clinical variants of vitiligo (%)			
Nondermatomal (44.6)	12.5 ± 3.2	9.7 ± 1.8	1.2 ± 0.2
Acrofacial (25.4)	14.4 ± 2.7	8.9 ± 1.2	1.6 ± 0.3
Mucosal (17)	10.5 ± 3.1	9.1 ± 2.1	1.1 ± 0.3
Focal (13)	11.0 ± 2.0	8.9 ± 1.7	1.2 ± 0.1
One-way ANOVA “*p*” value	0.000^**∗**^	**0.111 (NS)**	0.000^**∗**^
Disease status (%)			
Stable (36.2)	11.5 ± 2.7	9.3 ± 1.8	1.2 ± 0.3
Active (63.8)	13.0 ± 3.3	9.3 ± 1.7	1.4 ± 0.3
“*t*-” test *p* value	0.009^**∗**^	**1 (NS)**	0.0004^**∗**^
Family history (%)			
Yes (40)	12.9 ± 3.4	9.1 ± 1.6	1.4 ± 0.3
No (60)	12.1 ± 3.1	9.4 ± 1.8	1.3 ± 0.3
“*t*-” test *p* value	**0.1 (NS)**	**0.3 (NS)**	0.02^**∗**^
Male patients with SH (%)			
Yes (42)	14.2 ± 4.1	9.9 ± 2.0	1.45 ± 0.3
No (58)	10.9 ± 2.8	9.1 ± 2.0	1.2 ± 0.3
“*t*-” test *p* value	0.0004^**∗**^	**0.09 (NS)**	0.0008^**∗**^

Note: NS: not significant; ^*∗*^ *p* < 0.05, *n* = number of subjects, SD = standard deviation, and SH = social habits.
